# Novel *LRPPRC* compound heterozygous mutation in a child with early-onset Leigh syndrome French-Canadian type: case report of an Italian patient

**DOI:** 10.1186/s13052-020-00903-7

**Published:** 2020-09-24

**Authors:** Ettore Piro, Gregorio Serra, Vincenzo Antona, Mario Giuffrè, Elisa Giorgio, Fabio Sirchia, Ingrid Anne Mandy Schierz, Alfredo Brusco, Giovanni Corsello

**Affiliations:** 1 Department of Health Promotion, Mother and Child Care, Internal Medicine and Medical Specialties “G. D’Alessandro”. University Hospital “P.Giaccone”, University of Palermo, Piazza delle Cliniche, 2, 90127 Palermo, Italy; 2 Department of Medical Sciences, University of Torino, Via Santena 19, 10126 Torino, Italy; 3 Institute for Maternal and Child Health IRCCS Burlo Garofolo, Via dell’ Istria, 65, 34137 Trieste, Italy

**Keywords:** LSFC, Mitochondrial disease, Hypotonia - developmental delay, Whole-exome sequencing

## Abstract

**Background:**

Mitochondrial diseases, also known as oxidative phosphorylation (OXPHOS) disorders, with a prevalence rate of 1:5000, are the most frequent inherited metabolic diseases. Leigh Syndrome French Canadian type (LSFC), is caused by mutations in the nuclear gene (2p16) leucine-rich pentatricopeptide repeat-containing (*LRPPRC*). It is an autosomal recessive neurogenetic OXPHOS disorder, phenotypically distinct from other types of Leigh syndrome, with a carrier frequency up to 1:23 and an incidence of 1:2063 in the Saguenay-Lac-St Jean region of Quebec. Recently, LSFC has also been reported outside the French-Canadian population.

**Patient presentation:**

We report a male Italian (Sicilian) child, born preterm at 28 + 6/7 weeks gestation, carrying a novel *LRPPRC* compound heterozygous mutation, with facial dysmorphisms, neonatal hypotonia, non-epileptic paroxysmal motor phenomena, and absent sucking-swallowing-breathing coordination requiring, at 4.5 months, a percutaneous endoscopic gastrostomy tube placement. At 5 months brain Magnetic Resonance Imaging showed diffuse cortical atrophy, hypoplasia of corpus callosum, cerebellar vermis hypoplasia, and unfolded hippocampi. Both auditory and visual evoked potentials were pathological. In the following months Video EEG confirmed the persistence of sporadic non epileptic motor phenomena. No episode of metabolic decompensation, acidosis or ketosis, frequently observed in LSFC has been reported. Actually, aged 14 months corrected age for prematurity, the child shows a severe global developmental delay. Metabolic investigations and array Comparative Genomic Hybridization (aCGH) results were normal. Whole-exome sequencing (WES) found a compound heterozygous mutation in the *LRPPRC* gene, c.1921–7A > G and c.2056A > G (p.Ile686Val), splicing-site and missense variants, inherited from the mother and the father, respectively.

**Conclusions:**

We first characterized the clinical and molecular features of a novel *LRPPRC* variant in a male Sicilian child with early onset encephalopathy and psychomotor impairment. Our patient showed a phenotype characterized by a severe neurodevelopmental delay and absence of metabolic decompensation attributable to a probable residual enzymatic activity. *LRPPRC* is a rare cause of metabolic encephalopathy outside of Québec. Our patient adds to and broaden the spectrum of LSFC phenotypes. WES analysis is a pivotal genetic test and should be performed in infants and children with hypotonia and developmental delay in whom metabolic investigations and aCGH are normal.

## Introduction

Leigh syndrome, French-Canadian type (LSFC, MIM#220111) is a neurogenetic degenerative disorder caused by mutations in the nuclear gene leucine-rich pentatricopeptide repeat-containing (*LRPPRC*) localized on 2p16. It is phenotypically distinct from other types of Leigh syndrome, for the possible early occurrence of fatally severe metabolic crises [1]. As a result of a founder effect, this rare monogenic autosomal recessive mitochondrial disease is more prevalent in the Saguenay-Lac-St Jean (SLSJ) region of Quebec [2] with a carrier frequency up to 1:23 adults and an incidence of 1:2063 live births [2, 3]. *LRPPRC* is a multifunctional protein belonging to a family of pentatricopeptide repeat (PPR) proteins containing a canonical 35 residue repeat motif that confer an ability to recognize RNA substrates regulating several post-transcriptional processes such as RNA editing, RNA stability or RNA degradation [4, 5]. Most LSFC patients are homozygous for an A354V substitution that causes a decrease in the expression of the LRPPRC protein [6]. Biochemically, LSFC is characterized by a severe cytochrome-c oxidase (COX) deficiency in the brain and liver, while in other tissues, such as kidneys, skeletal muscle, and heart, COX activity is affected to a lesser extent (50–80% residual activity) [1, 2]. In recent years, novel disease-causing mutations have been reported from some unrelated families outside the French-Canadian population [6]. Early onset, from birth to 5 months, has been described as characterized by neonatal neurological findings including beyond hypotonia, transient tachypnea, poor sucking, tremor, along with acute metabolic decompensation including acidosis and ketosis, described as frequently fatal, and stroke-like episodes [1, 7]. In survivors a severe neurological impairment is described including: dystonia, ataxia, dysphagia, strabismus and seizures. Brain imaging has evidenced in some patients, cerebral malformations (cerebellar hypoplasia, gyral abnormalities, and hippocampal abnormalities), leukoencephalopathy, and progressive degenerative involvement of brainstem and basal ganglia [6, 8]. Consistently dysmorphic cranio-facial features as prominent forehead, anteverted nares, arched eyebrows hypertelorism, with varying degrees of midfacial hypoplasia have been described [2]. Moreover, polysyndactyly, complex congenital heart disease, hypertrophic cardiomyopathy, hypospadias and anteriorly placed anus have been sporadically reported [6].

## Patient presentation

Since our patient was born preterm, in this report we have referred to both a chronological age (CrA) and a corrected age for prematurity (CA), considering the difference of 78 days to reach the 280 days length of full-term pregnancy.

**Fig. 1:**
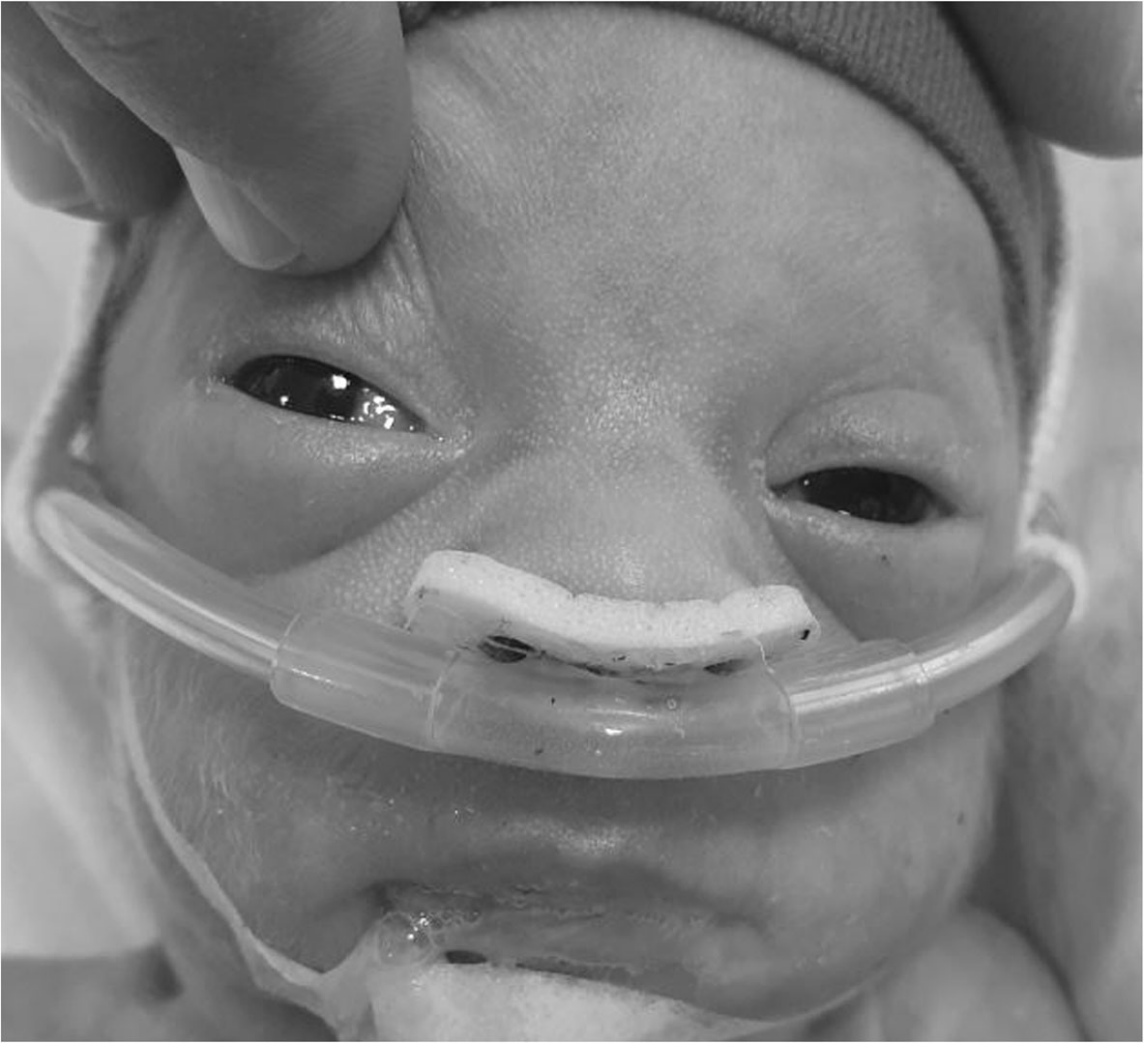
Neonatal dysmorphic facial features: prominent forehead, hypertelorism, broad nasal bridge, flattened philtrum, thin upper lip. Right exophoria is present

**Fig. 2:**
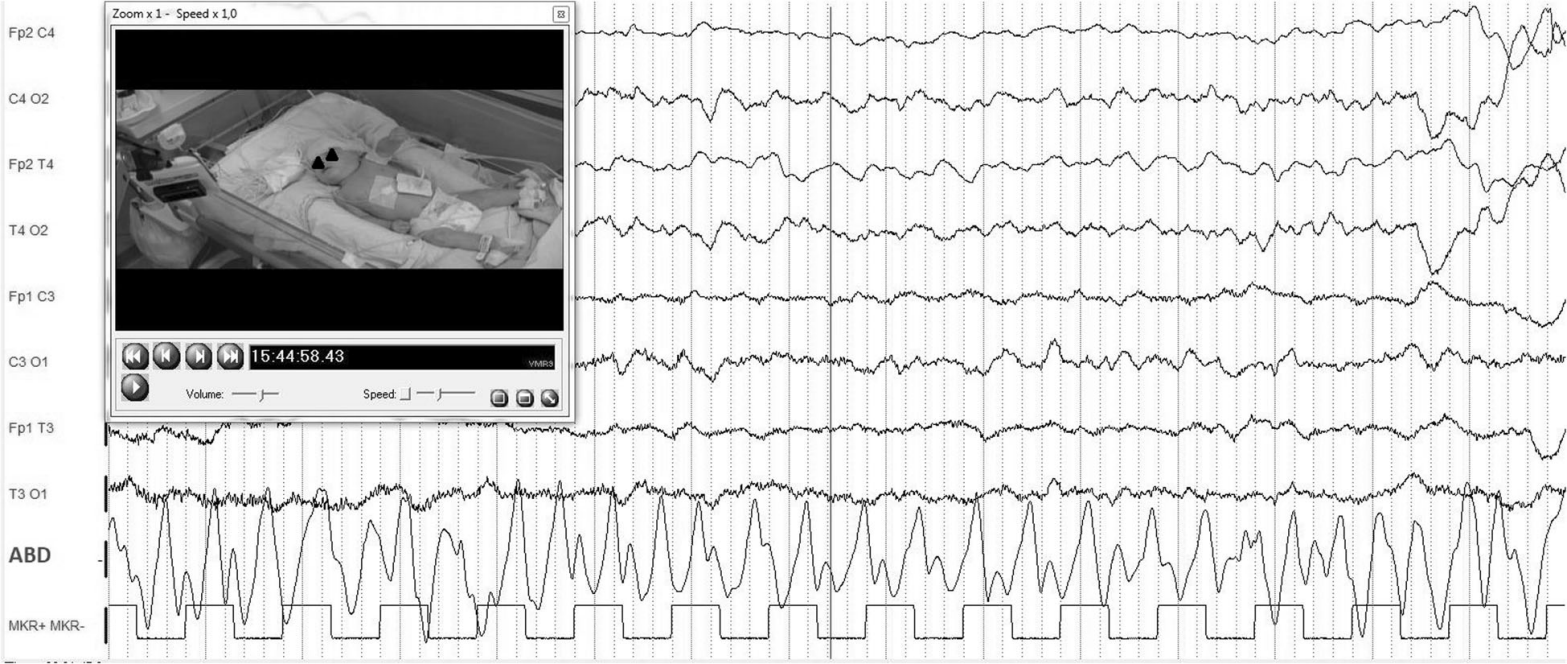
Awake video-EEG at 2.5 months CA: spontaneous motor paroxysm involving left harm without concomitant EEG discharge

**Fig. 3:**
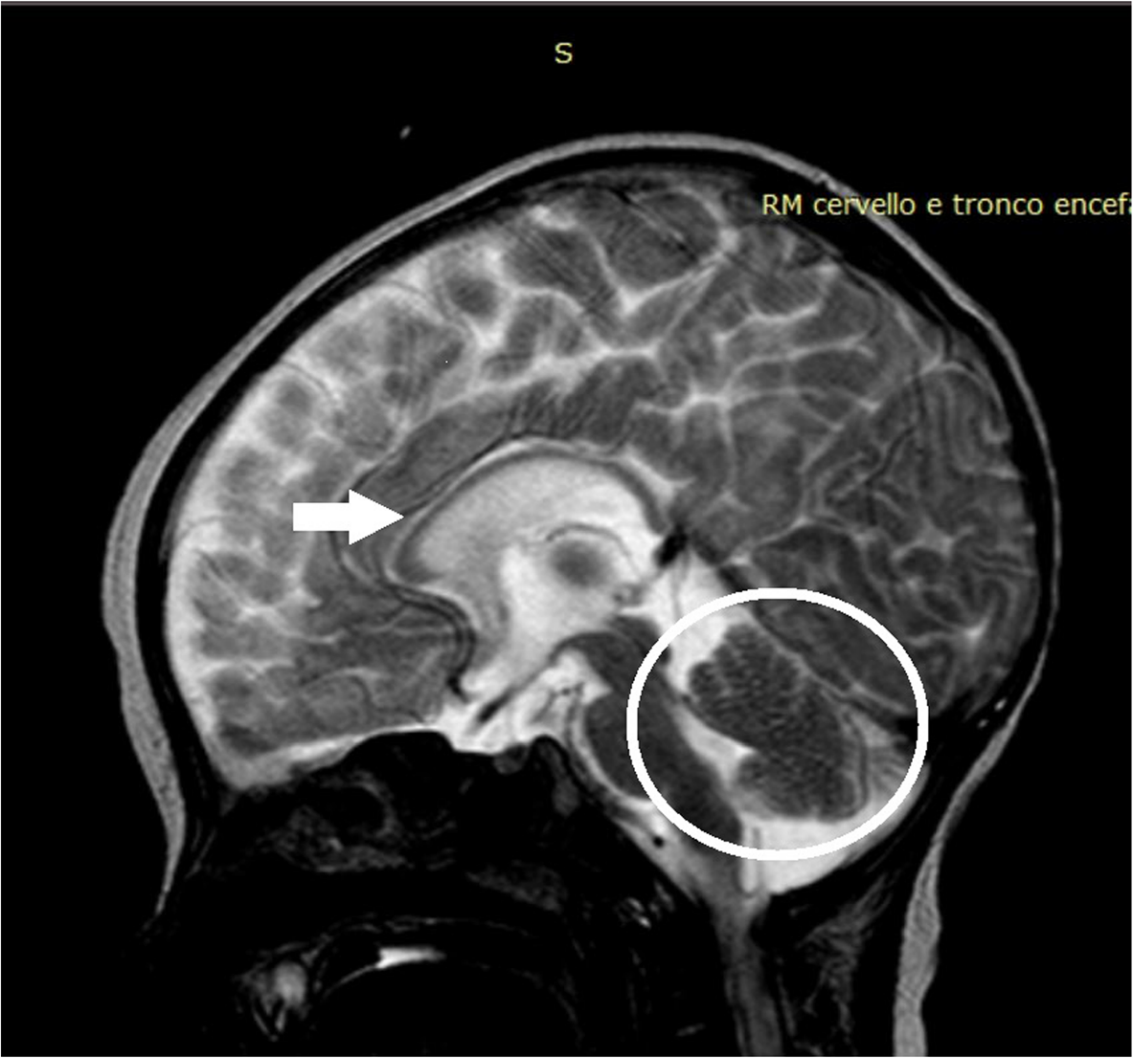
Brain MRI T2 sagittal image: mild diffuse cerebral atrophy, hypoplasia of the corpus callosum (white arrow), cerebellar vermis hypoplasia (white circle)

**Fig. 4:**
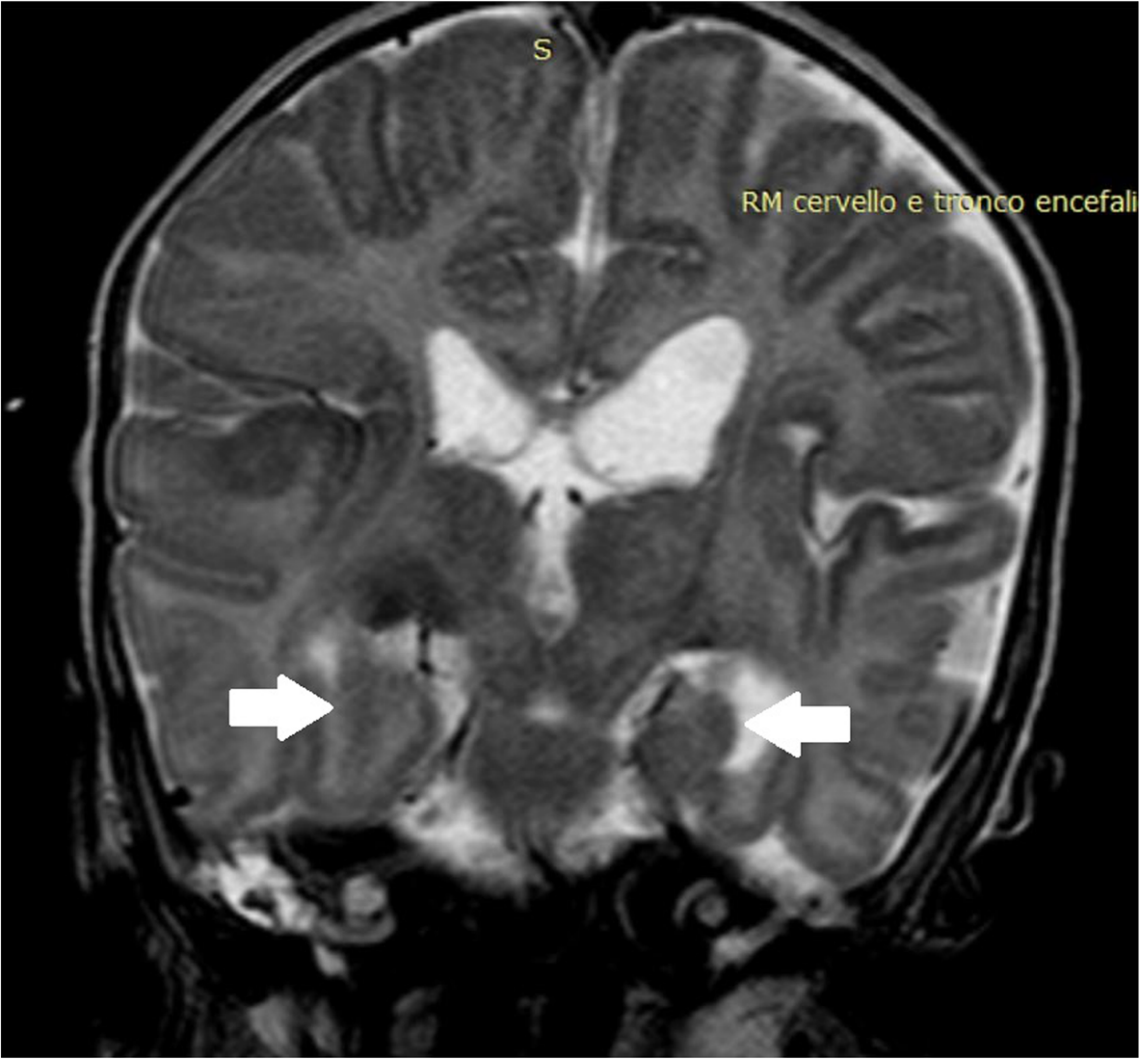
Brain MRI T2 coronal image: incomplete bilateral hippocampal inversion (white arrows) and left lateral ventricle dilatation

**Fig. 5:**
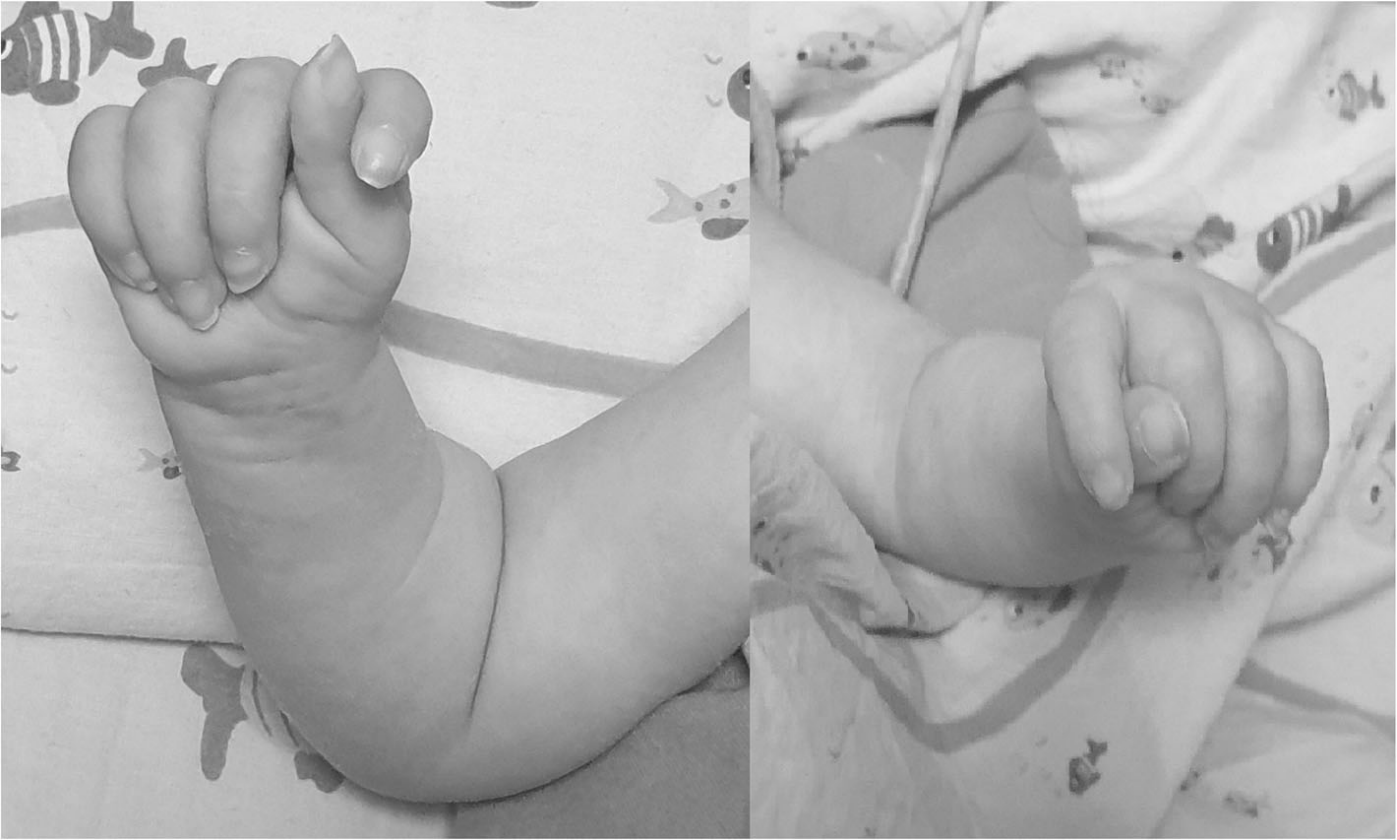
Patient 14 months CA: Bilateral adducted thumbs

The proband was the second child of healthy and nonconsanguineous Italian parents from Sicily. He was born preterm, at 28 + 6/7 weeks after a naturally conceived pregnancy, by cesarean section for premature rupture of membranes, chorioamnionitis and fetal distress. Apgar scores were 7 and 7, at 1 and 5 min, respectively. At birth, his weight was 1360 g (80th centile), length 40 cm (86th centile), and occipitofrontal circumference (OFC) 28.4 cm (89th centile). On physical examination, he showed dysmorphic features: prominent forehead, hypertelorism, broad nasal bridge, anteverted nares, flattened philtrum, thin upper lip, and high-arched palate. Neurological examination revealed, marked generalized hypotonia with presence of deep tendon reflexes, depressed neonatal reflexes, poor spontaneous motility, right exophoria, disconjugated eye movements and weak cry (Fig. 1). Brain ultrasound (US) showed normal corpus callosum and mild dilatation of the left lateral ventricle. He had poor respiratory adaptation with tachypnea needing non-invasive ventilatory support from birth to 3 months CrA (15 days CA). From birth repeated Video-EEG monitoring showed sporadic spontaneous as well as provoked clonic and spontaneous motor paroxysms involving limbs, without concomitant EEG discharge (Fig. 2). He necessitated of frequent oropharyngeal aspirations due to swallowing dysfunction, an early sign of incoordination of sucking-swallowing and breathing. These findings suggested, in relation to failure to thrive and frequent episodes of reflux associated with apnea, to convert to a nasogastric tube feeding regimen until insertion, at 4.5 months CrA, of a percutaneous endoscopic gastrostomy (PEG). At 5 months of CrA, corresponding to 2.5 months CA, a brain MRI revealed a mild diffuse cerebral atrophy, hypoplasia of the corpus callosum, cerebellar vermis hypoplasia (Fig. 3) and bilateral incomplete hippocampal inversion (Fig. 4). Visual evoked potentials (VEP) recorded to binocular flash stimulation showed a delayed P100 latency (240 msec in right hemisphere, 270 msec in left hemisphere), and brainstem auditory evoked potentials (BAEP) showed increased hearing threshold (60 dB) in the right ear and absence response even at high-intensity stimulation (110 dB) in the left ear. Extended newborn screening for inherited metabolic disorders and aCGH were negative. Thus, on the basis of the clinical evolution suggesting a mitochondrial encephalopathy, a further metabolic diagnostic work-up was carried on. Plasma amino acids, acylcarnitines and biotinidase were normal, while urinary organic acids analysis showed marked increase of 4-OH phenylacetic acid, along with a moderate increase of phenylpyruvic and mild increase of ethylmalonic and fumaric acids. Thus, at 5 months CA, WES analysis was performed. The genomic sequencing, followed by direct sequencing, found and confirmed that the patient carried a compound heterozygous mutation in the *LRPPRC* gene, c.1921–7A > G and c.2056A > G (p.Ile686Val), splicing-site and missense variants, inherited from the mother and the father, respectively. The mutation of maternal origin and has not hitherto been described in the literature. Therefore, based on current knowledge, the variant can be classified as likely pathogenic (class IV). The second variant of paternal origin consisted in a replacement of an adenine with a guanine in position 2056 of the codon 686, determining the substitution of isoleucine with valine. This variant has not been described in the literature too, and according to current knowledge, can be classified of uncertain significance (class III). Parents refused any further invasive investigation for the execution of functional studies that could demonstrate COX activity deficit. Thus, to assess the functional effect of mRNA transcripts, we performed a reverse transcription-polymerase chain reaction (RT-PCR) that demonstrated the deletion of 45 base pairs in the exon 19 with a likely deletion of 15 amino acids in the encoded protein. He is currently 14 months CA, his weight is 5.500 g (− 5.4 SD), length 65 cm (− 5.2 SD) and OFC 40.6 cm (− 4.6 SD). Owing to the severe generalized hypotonia he has to lie in bed with nasal cannula O2 supplementation. He shows only sporadic movements of head rotation without focusing on a nearby object. Neither vocalization nor blinking reaction to bell and rattle are present. He is unable to move his limbs and maintains adducted thumbs in both hands (Fig. 5). Nutritional supply with protein hydrolysate, MCT, Vitamin supplements, L-carnitine and glycopyrrolate for drooling are provided by PEG. However, he has never experienced to date episodes of acute ketosis, glycemic derangements, or any acute stroke like events. A home rehabilitation treatment has been started.

## Discussion and conclusions

In 1994, Hou first identified the *LRPPRC* gene, which encodes for a protein containing 35 amino acid repeat motifs [9], that conferring an ability to recognize RNA substrates, regulate a number of post-transcriptional processes such as RNA editing, stability or degradation [4]. Next generation sequencing, including WES, Whole Genome Sequencing and RNA sequencing, has revolutionized the diagnostic approach of rare genetic disorders, making it possible to undertake a phenotyping driven by the results from genetic markers, called “reverse phenotyping” approach [10, 11].

In our patient we adopted a “reverse phenotyping” approach, since WES analysis through the identification of novel LRPPRC compound heterozygous mutations, made it possible to refine the patient phenotype and the diagnosis of LSFC.

We identified a novel pathogenic compound heterozygous *LRPPRC* mutation in a male Italian child from Sicilian parents, consisting of a maternal splicing site (class IV) and a paternal missense (class III) variant. Thus, our proband did not carry, the most reported French-Canadian founder mutation. However, his clinical features resembled LSFC in many aspects including severe global neurodevelopmental impairment, generalized hypotonia, dystonia, dysphagia, exophoria, visual and hearing impairment and post-natal growth deficiency. In our patient brain anomalies and reduced neuronal cell proliferation leading to cortical atrophy, hypoplasia of corpus callosum, cerebellar vermis hypoplasia, left lateral ventricular dilatation, unfolded hippocampi, have been evidenced by brain MRI. We suppose that the described involvement of basal ganglia, and midbrain, reported in some patients was not still present probably owing to the younger age [6]. Neither episodes of severe (sometimes fatal) lactic acidosis, nor ketosis or hyperglycemia were present in our patient, whose lactic acid levels maintained from birth levels < 2.5 mmol/l. Urinary organic acids analysis showed marked increase of 4-OH phenylacetic acid, along with a moderate increase of phenylpyruvic and mild increase of ethylmalonic and fumaric acids. These results were not suggestive of mitochondrial deficit. Since in our patient we have not performed Western blot analyses for dosing protein levels of subunits of mitochondrial respiratory chain complexes, we can only speculate that a less severe compound heterozygous mutation could have avoided, to date, a fatal metabolic crisis, as well as the onset of left ventricle cardiomyopathy frequently found by others [6]. Indeed, these last deleterious effects have been found to be more frequently related to the *LRPPRC* A354V founder mutation, responsible for an increased mitochondrial vulnerability and nutrient-induced cytotoxicity [12]. Moreover, different dose effects could also result from functional differences in gene expression depending on the parental origin of the single mutation, as recently described in other neurodevelopmental disorders [13].

A pathogenic link between LSFC and Neurofibromatosis type 1 (NF1), a clinically heterogeneous, neurocutaneous genetic disorder [14], has been recently found studying the RNA granules. The RNA granules play a key role in regulating de novo protein synthesis in a temporal and spatial manner by microtubule-dependent, motor protein-driven transport of mRNA cargoes to subcellular sites of requirement [15]. The tubulin binding domain of NF1 is a binding partner of the LRPPRC protein and both proteins complex with Kinesin 5B, Staufen1, hnRNP A2, and Myelin Basic Protein mRNA, likely in RNA granules. These findings provide clues to how loss or mutation of *NF1* and *LRPPRC* may contribute to the wide range of clinical manifestations reported in both condition [16]. Despite significant advances in understanding the molecular genetics of LSFC, the pathogenic mechanisms (precise molecular ones through which *LRPPRC* stabilizes mitochondrial transcripts) underlying this severe and unpredictable disease remain unclear. In most mitochondrial diseases, impaired capacity to generate ATP is believed to be the main culprit and mitochondria play a central role in other numerous vital processes (including triggering of programmed cell death), all of which could contribute to dysfunction and death, particularly when cells are faced with stressful conditions. On the other hand, a compensatory mechanism to preserve ATP level and consisting in the activation of the mTORC1 pathway and its downstream target HIF-1α/PDHK1n LSFC fibroblasts has been recently described [17].

Effective treatment strategies for these patients are lacking. Recent progress achieved in pharmacogenomics might provide an effective treatment targeting potential consequences of COX deficiency such as impaired electron transport chains flux, oxidative stress, mitochondrial cell death signaling, and accumulation of toxic metabolites with interesting concerns about the nature of the diets, particularly excess fat intake, as well as on the use of antioxidants in patients with LSFC and, possibly, other COX defects have been raised. Indeed, Burelle et al. have demonstrate the protective effect of compounds (methylene blue and dinitrophenol) that promote flux through the electron transport chain independent of phosphorylation, and that modulate fatty acid (L-carnitine) or Krebs cycle metabolism (propionate), while antioxidants (resveratrol, idebenone, Nacetyl cysteine) exacerbate palmitate plus lactate-induced cell death [12].

In this report we have described the clinical and genetic aspects of a novel compound heterozygous mutation of *LRPPRC* in an Italian male child with early onset LSFC characterized by severe neurologic involvement and facial dysmorphic features. The absence of early metabolic crisis or relevant lactic acidosis broaden the spectrum of phenotypes of LSFC. Our patient is one of the few reported outside of the French-Canadian population and the first, to our knowledge, observed in Italy. Integrated and family centered model of care for potential end-of-life pathologies should implement protocols that reduce unreasonable therapeutic obstinacy [18] and offer parents acceptance and commitment based coping strategies [19]. WES analysis, a pivotal genetic test for early diagnosis, prognostic definition and accurate genetic counselling, should be performed in infants and children with hypotonia and developmental delay in whom metabolic investigations are normal and chromosomal aneuploidy, microdeletions and microduplications have been excluded by aCGH. Moreover, it has been recently proposed as a powerful tool for the diagnostic evaluation of critically ill infants with suspected monogenic disorders in the Neonatal and Pediatric Intensive Care Units and its use has a notable effect on clinical decision-making process [20].

## Supplementary information

**Additional file 1.**

## Data Availability

The clinical data used during the current study are available from the corresponding author on reasonable request.

## Abbreviations

aCGH: Array Comparative Genomic Hybridization
LSFC: Leigh Syndrome French Canadian type
*LRPPRC*: Leucine-rich pentatricopeptide repeat-containing
WES: Whole-Exome Sequencing
COX: Cytochrome-c oxidase
CrA: Chronological age
CA: Corrected age for prematurity
OFC: Occipitofrontal circumference
US: Ultrasound
PEG: Percutaneous endoscopic gastrostomy
VEP: Visual evoked potentials
BAEP: Brain auditory evoked potentials
dB: Decibel
RT-PCR: Reverse transcription-polymerase chain reaction
SD: Standard deviation
NF1: Neurofibromatosis type1
