# Supplementary material for: Novel LRPPRC compound heterozygous mutation in a child with early-onset Leigh syndrome French-Canadian type: case report of an Italian patient
Source: Ital J Pediatr. 2020 Sep 24;46:140. doi: 10.1186/s13052-020-00903-7 (PMC7517646; doi:10.1186/s13052-020-00903-7)
Supplement: Supplementary file 1 — Additional file 1. [file 13052_2020_903_MOESM1_ESM.docx]

**Patient 28+^6/7^ gestational age**

**CA= Corrected Age for Prematurity**

Admission to UTIN

Brain US - videoEEG

**CrA=Chronological Age**

Birth

**mos= Months**

At term CA/ 2,5 mos CrA

14 mos CA/16.5 mos CrA

Hypotonia

Developmental Delay

Severe growth impairment

PEG - MRI- VEP- BAEP - WES

2,5 mos CA/5 mos CrA

aCGH
